# Cooking With Liquefied Petroleum Gas or Biomass and Quality of Life: Results From a Randomized Controlled Trial

**DOI:** 10.1155/ina/9936046

**Published:** 2026-04-15

**Authors:** Suzanne M. Simkovich, Shakir Hossen, Kendra N. Williams, Josiah L. Kephart, Magdalena Fandiño-Del-Rio, Carla Tarazona-Meza, Dina Goodman, Mitra Moazzami, Victor G. Dávila-Román, Kyle Steenland, Laura Nicolaou, David Dowdy, Kirsten Koehler, William Checkley, Cardiopulmonary Outcomes and Household Air Pollution (CHAP) Trial Investigators

**Affiliations:** 1 Division of Healthcare Delivery Research, MedStar Health Research Institute, Hyattsville, Maryland, USA; 2 Division of Pulmonary and Critical Care Medicine, Georgetown University, Washington, D.C., USA; 3 Center for Global Non-Communicable Disease Research and Training, School of Medicine, Johns Hopkins University, Baltimore, Maryland, USA; 4 Division of Pulmonary and Critical Care, Johns Hopkins University School of Medicine, Baltimore, Maryland, USA; 5 Department of International Health, Bloomberg School of Public Health, Johns Hopkins University, Baltimore, Maryland, USA; 6 Department of Environmental and Occupational Health, Dornsife School of Public Health, Drexel University, Philadelphia, Pennsylvania, USA; 7 Department of Environmental Health and Engineering, Bloomberg School of Public Health, Johns Hopkins University, Baltimore, Maryland, USA; 8 Biomedical Research Unit, A.B. PRISMA, Lima, Peru; 9 Cardiovascular Division, Department of Medicine, Washington University, St. Louis, Missouri, USA; 10 Department of Environmental Health, Rollins School of Public Health, Emory University, Atlanta, Georgia, USA; 11 Department of Epidemiology, Bloomberg School of Public Health, Johns Hopkins University, Baltimore, Maryland, USA

## Abstract

Household air pollution from biomass fuel combustion affects approximately 36% of the world’s population and is associated with negative health, environmental, economic, and social impacts. Although randomized controlled trials (RCTs) designed to reduce household air pollution have largely focused on health and environmental outcomes, evidence regarding their impact on quality of life (QOL) and household financial status remains limited. We conducted an RCT to evaluate the impact of a liquefied petroleum gas (LPG) intervention on health outcomes among 180 women aged 25–64 years who were the primary cooks in their households. We randomly assigned half of the participants to receive an LPG stove, continuous fuel supply for 12 months, and behavioral reinforcement. The other half continued usual cooking practices. We assessed QOL as a secondary outcome using the Short Form 36 Version 2 (SF-36-v2) and the EuroQol-5D-3L (EQ-5D). We also asked participants to report any perceived change in their household financial status 12 months after randomization. A total of 179 women (mean [±SD] age 48.3 ± 10.1 years) completed the surveys. In intention-to-treat analyses, participants in the intervention arm had similar SF-36 scores at 12 months compared with controls (mental component score: 48.3 vs. 50.9, *p* = 0.59; physical component score: 53.1 vs. 47.6, *p* = 0.08) and EQ-5D health state scores were also similar (67.0 vs. 71.7, *p* = 0.05). Exposure–response analyses did not reveal a significant association between personal PM_2.5_ exposure and QOL scores. However, a significantly larger proportion of women in the intervention group reported feeling financially better off (42% vs. 13%; *p* < 0.001) and higher income (33% vs. 9%; *p* < 0.001) at 12 months compared with baseline. Although the intervention did not result in measurable differences in QOL scores, participants perceived meaningful improvements in their household financial status. Future research should explore the broader QOL, social, and economic benefits of transitioning to cleaner cooking fuels.

## 1 | Introduction

Wood, dung, agricultural crop waste, and coal are used as the primary sources of energy in approximately 36% of households worldwide [1]. The resulting household air pollution (HAP) is associated with a wide range of adverse health, environmental, social, and economic outcomes. In 2021, an estimated 3.11 million deaths and 111 million disability-adjusted life years lost were attributed to HAP [2]. HAP contributes to a wide range of adverse health outcomes, including chronic obstructive pulmonary disease, cardiovascular disease, cancer, and pneumonia [3–7]. Chronic conditions, many of which are linked to biomass smoke exposure, are associated to premature disability, increased medical costs, poverty, and a lower quality of life (QOL) in low- and middle-income countries (LMICs) [8, 9].

Cross-sectional studies have found that individuals who use biomass stoves report lower QOL compared to those who use cleaner fuels [10–13]. Similarly, exposure to higher concentrations of ambient air pollution has been associated with worse QOL [14–16]. However, the effects of transitioning from biomass to cleaner fuels on measured QOL outcomes or household financial status have yet to be rigorously evaluated in randomized controlled trials (RCTs) [17, 18].

Cooking and heating with biomass fuels are time-consuming and labor-intensive activities. Typically, a female household member is responsible for fuel collection, often walking long distances across potentially hazardous terrain [19]. Women may also face the risk of violence while collecting fuel [20]. Once collected, it must be prepared: Wood is chopped, crop waste is dried, and dung is shaped into patties and dried [11]. During cooking, fuel must be fed continuously into the fire, which burns inefficiently and releases fine particulate matter (PM_2.5_, particles ≤ 2.5 *μ*m in aerodynamic diameter), carbon monoxide (CO), black carbon (BC), and nitrogen dioxide (NO_2_) among other toxic gases [20]. These pollutants can be inhaled and leave visible soot and dirt on the walls, ceilings, and floors of the household cooking areas [21].

Switching from biomass fuels to cleaner fuels such as liquefied petroleum gas (LPG) may improve QOL and household wealth. Previous studies have shown that users of cleaner, more efficient fuels have more time for leisure and income-generating activities compared to those who rely on biomass [11, 22, 23]. These findings are consistent with broader evidence that socioeconomic status is positively associated with QOL in LMIC settings [24–26]. Moreover, reduced exposure to HAP may lower the risk of chronic illness, which would be expected to improve QOL [14, 27].

In the Cardiopulmonary Outcomes and Household Air Pollution (CHAP) trial, we evaluated the QOL among participants who switched from cooking primarily with biomass to LPG [28]. We hypothesized that intervention participants would report higher QOL and quality-adjusted life years (QALYs) compared with controls.

## 2 | Methods

### 2.1 | Study Setting and Design

The CHAP trial was an RCT conducted in rural communities of Puno, Peru. The trial enrolled 180 women aged 25–64 years who used biomass fuels daily for cooking. Participants were randomly assigned to receive a free LPG stove, a continuous fuel supply, and education with behavioral reinforcement promoting exclusive LPG use for 1 year (*n* = 90), or to continue their usual cooking practices (*n* = 90) [28].

The primary endpoints of the CHAP trial, as published elsewhere, included blood pressure, peak expiratory flow, and respiratory symptoms [18, 28]. QOL and health utility assessments, measured by the EuroQol-5D-3L (EQ-5D) and the Rand Short Form 36 Version 2 (SF-36-v2), were prespecified secondary outcomes and therefore exploratory in nature [28]. Study design, data collection, analysis, and other results are described in detail elsewhere [18, 29–35].

### 2.2 | Data Collection

QOL surveys were administered in person at baseline and at 3, 6, and 12 months postrandomization; proxy responses were not permitted. A sociodemographic survey was administered at baseline and again at 12 months postrandomization. The 12-month sociodemographic survey asked participants about their current economic situation and income relative to the period before the intervention (see Online Supplement for details). All survey data were collected electronically by trained field workers fluent in Spanish and Aymara using the Research Electronic Data Capture (REDCap) software [36, 37].

The EQ-5D is a standardized, preference-based instrument comprising five domains: mobility, self-care, usual activities, pain/discomfort, and anxiety/depression [38]. For each domain, participants choose one of three levels of severity: no problems, some problems, or extreme problems. Domain-level results are reported as the proportion of participants selecting each response level. An index score (health state) was calculated for each participant using a country-specific value set. Because no value set is currently available in Peru, we used a Chilean value set, selected due to cultural and economic similarities between Chile and Peru [39, 40]. QALYs were derived from these health state values. The Spanish (Peru) version of the EQ-5D was administered under license from the EuroQol Research Foundation to all participants at baseline and at 3, 6, and 12 months postrandomization [38].

The EQ-5D data were scored using R statistical software as previously described [41]. The recall period reflected the participant’s health status on the day of administration [38]. The minimal important difference (MID) was 0.04 points, which was based on an estimate for the general population of Uruguay, the most comparable country to Peru with available MID estimates [42].

The SF-36-v2 is another standardized questionnaire assessing eight domains: physical functioning, bodily pain, role limitations due to physical health problems, role limitations due to personal or emotional problems, general mental health, social functioning, energy/fatigue, and general health perceptions [43]. It yields two summary scores: the physical component score (PCS) and the mental component score (MCS). SF-36-v2 responses can also be converted into the SF-6D, a preference-based measure used to estimate health utility and QALYs.

We used the Spanish version of SF-36-v2 [44, 45]. Due to delays in licensing agreements, SF-36 -v2 administration began in February 2018, resulting in missing baseline and early follow-up data; however, complete data were obtained at the 12-month assessment. Analyses were restricted to participants with complete data. SF-36-v2 was scored using ProCore Software. In the absence of country-specific preference weights, we used the United States value set. The recall period for this instrument was 4 weeks [43]. The standardized MID for the SF-6D is 0.03 points [46].

### 2.3 | Exposure and Stove Use Assessments

We measured 48-h personal exposures to PM_2.5_ and CO concentrations at baseline, 3, 6, and 12 months postrandomization using the Enhanced Children’s MicroPEM (ECM, RTI International, Research Triangle Park, NC, United States) and the EasyLog Carbon Monoxide data logger with USB (EL-USB-CO Lascar Electronics, Erie, PA, United States), respectively. We also monitored stove use by installing LabJack Digit-TL temperature sensors (LabJack Corporation, Lakewood, CO, United States) on all biomass-burning and LPG stoves in the household [47].

Detailed descriptions of exposure assessment and stove use monitoring are described elsewhere [23, 30]. The intervention reduced PM_2.5_ levels. Compared with controls, intervention households had lower postrandomization kitchen -area PM_2.5_ concentrations (58 vs. 1246 *μ*g/m^3^) and personal exposures (30 vs. 98 *μ*g/m^3^), both statistically significant (*p* < 0.001). Additionally, 23% of intervention kitchens and 78% of participants had PM_2.5_ levels below 35 *μ*g/m^3^, compared to 0% and 13% in the control group, respectively.

### 2.4 | Biostatistical Methods

The primary analytical objective of this analysis was to evaluate the effect of the LPG intervention on QOL outcomes and perceived household wealth. Using an intention-to-treat analysis, we compared QOL measurements at baseline and at 3, 6, and 12 months postrandomization between the intervention and control participants using *t*-tests to compare continuous variables and either chi-square tests or Fisher exact tests for categorical or dichotomous variables between groups at each time point. We conducted subgroup analyses by age (≥ 50 or < 50 years), LPG stove ownership at baseline, mean systolic blood pressure (≥ 100 or < 100 mm Hg), mean total daily cooking time (≥ 190 or < 190 min), mean surveyed daily cooking time (≥ 180 or < 180 min), education (> 5 or ≤ 5 years), and occupation (farmer/laborer, housewife, or merchant) [18, 23]. We also conducted exposure–response analyses with QOL scores as the response and personal exposure to PM_2.5_ as the exposure. For this analysis, we used a generalized additive model for QOL as a function of a smoothing spline for personal exposure to PM_2.5_ and a random intercept for each individual, adjusting for age using a smoothing spline, body mass index, presence of a chimney for the cookstove, number of people who sleep in the house, wealth index, ownership of pigs or dogs in the household, years of education, prior LPG use, and participation in the national LPG subsidization program (Fondo de Inclusión Social Energético or FISE for its acronym in Spanish). Analyses were conducted using R version 4.1.2 (alias Bird Hippie) and the packages *EQ5D*, *tidyverse*, *nlme*, *itsadug*, *tableone*, and *moments* [48].

## 3 | Results

### 3.1 | Participant Characteristics

A total of 180 participants completed the sociodemographic questionnaire at baseline (Table 1) and 179 completed it at 12 months postrandomization. One participant withdrew before the 12-month assessment. The EQ-5D was completed by 180 participants at baseline, 3 months, and 6 months, and by 179 at 12 months (Table 2). The SF-36-v2 was completed by 16 participants at baseline, 61 at 3 months, 93 at 6 months, and 179 at 12 months. The mean (±SD) age at baseline was 48.3 ± 10.1 years.

### 3.2 | Intention-to-Treat Analyses

In Figure 1, we show mean scores and corresponding 95% confidence intervals for EQ-5D and SF-36-v2 by assignment arm and visit. Mean QOL scores were consistent over time, with no differences between trial arms at any time point that exceeded the MID or reached statistical significance. At 12 months postrandomization, participants in the intervention arm had comparable mean SF-36-v2 scores to those in the control arm (MCS 48.3 vs. 50.9 points, *p* = 0.59; PCS 53.1 vs. 47.6 points, *p* = 0.08). For the EQ-5D, mean health state scores were slightly higher in the control group than in the intervention group at 12 months (71.7 vs. 67.0 points; *p* = 0.05).

### 3.3 | Subgroup Analyses

We did not find statistically significant differences between arms in any EQ-5D scores (Figure 2) or SF-36-v2 scores across predefined subgroups (Figure 3), and no differences exceeded MID thresholds. Intervention participants aged ≥ 50 years reported slightly higher scores across all components of the SF-36-v2 and EQ-5D, and those who had a total daily cooking time of ≥ 190 min had slightly higher QOL scores on all components except the mental health component of the SF-36-v2. However, none of these differences were statistically significant.

### 3.4 | Exposure–Response Analyses

Exposure–response analyses showed no consistent association between personal PM_2.5_ exposure and QOL or health utility measures (Figure 4). Comparing the 90^th^ percentile (173.2 *μ*g/m^3^) with the 10^th^ percentile (12.5 *μ*g/m^3^) of PM_2.5_ exposure suggested lower EQ-5D health state scores at higher exposures; however, this association was not statistically significant and did not exceed the EQ-5D MID. Similarly, higher PM_2.5_ exposure was associated with lower SF-36-v2 MCS and SF-6D scores, but these differences were not significant and did not exceed the MID.

### 3.5 | Change in Financial Status

At 12 months postintervention, a higher proportion of intervention participants reported feeling financially better off relative to baseline than participants in the control arm (42% vs. 13%; *p* < 0.001) (Table 3). Similarly, a greater proportion of intervention participants reported higher income relative to preintervention compared with controls (33% vs. 9%; *p* < 0.001). No intervention participants reported feeling financially worse off or lower income compared with baseline, whereas 3% of control participants reported worse financial status or lower income. Despite these perceived improvements, overall income levels at trial completion did not differ between arms.

## 4 | Discussion

In this randomized trial, we did not find differences in QOL between intervention participants using LPG and controls cooking with biomass, despite substantial and well-documented reductions in HAP exposures [29]. Similarly, exposure–response analyses did not identify significant differences in QOL between participants with lower versus higher levels of personal exposure. In contrast, intervention participants reported meaningful improvements in perceived household financial status at 12 months postrandomization. In addition, our study provides valuable reference data on QOL and health utility for a rural, low-income South American population, which may inform future economic evaluations of other interventions.

The absence of observed differences in QOL is consistent with the primary health outcomes of the CHAP trial [18]. We previously reported no significant differences between intervention and control participants in blood pressure, peak expiratory flow, or respiratory symptoms [18], nor did we find associations between these outcomes and household air pollutants in exposure–response analyses [18]. Nonetheless, we hypothesized that improvements in QOL would accompany other observed effects of the intervention, such as improved air quality, less time spent collecting and preparing biomass fuel, reduced time spent cooking with LPG, increased time for rest and sleep, and greater consumption of hot meals at lunchtime with LPG stove use [22, 23, 31]. The observed improvement in perceived financial status is likely attributable to the provision of clean fuels, which reduced time spent on fuel collection and cooking, allowing participants to reallocate time to other productive activities. We acknowledge that financial status is difficult to measure in this population, as currency and reported monthly income do not necessarily reflect overall wealth. Instead, ownership of animals and other goods may be more accurate indicators of financial status.

Our study contributes to a growing but mixed literature on the relationship between HAP and QOL outcomes. Prior studies have largely been cross-sectional and primarily focused on ambient air pollution or secondhand smoke rather than HAP [14, 49–52]. Results from these studies have been inconsistent, with some reporting improvements in specific SF-36 and EQ-5D domains, and others showing no association [14, 49–52]. Substantial heterogeneity exists across studies, many of which rely on self-report or other less reproducible measures [14, 49–52]. Evidence linking HAP directly to QOL is even more limited. For example, Alexander et al. [11] used the St. George’s Respiratory Questionnaire (SGRQ) to evaluate the effect of reduced CO exposures on respiratory QOL 1 year after distributing wood cookstoves with a chimney. They found that lower CO levels were associated with greater QOL, reflected on all domains of the SGRQ. In contrast, Checkley et al. did not find an association between HAP exposures and SGRQ scores [18]. Our results, which were generated using standardized and validated instruments and collected within the context of a clean fuel intervention that successfully reduced HAP, align with the latter findings, demonstrating no significant associations between exposures and QOL.

Several factors may explain the lack of observed improvement in QOL following the intervention. First, participants were generally healthy at baseline, resulting in relatively high QOL and limited potential for measurable improvement. Second, the 12-month intervention period may have been too short to produce meaningful changes in QOL. There is also no consensus in the literature on the optimal time frame for interventions to improve QOL [53, 54]. Moreover, it remains unclear how long clean fuels must be used to improve QOL or produce measurable health benefits. Finally, QOL is affected by multiple aspects of a person’s life. Notably, 92% of study participants were in the poorest two quintiles of wealth according to a national survey in Peru [18]. A transition to cleaner fuels alone may not be enough to produce improvements on broad measures of QOL or health utility, given the persistent socioeconomic challenges participants face.

Our analysis has several strengths. First, participants in the intervention arm experienced lower personal exposures to household air pollutants and demonstrated high compliance with LPG stove use [29]. Second, this study provides reference values for mean QOL and health utility among rural South American populations, which may inform future economic evaluations of interventions in similar settings. Finally, our locally developed survey showed perceived improvements in wealth and income, suggesting that the intervention may have positively affected the economic well-being of households, even if such improvements were not fully captured by standard QOL instruments [55, 56].

Despite the strengths, there are some potential shortcomings. First, this trial was not designed to detect changes in QOL, which were prespecified as secondary outcomes. The trial was powered to the primary outcomes (blood pressure, peak expiratory flow, and respiratory symptoms) as described elsewhere [18]. Second, the SF-36-v2 and EQ-5D instruments have not been formally validated or normed in our study population, limiting comparisons with reference populations. Although the SF-36-v2 has been validated in upper-middle-income countries and EQ-5D validation is ongoing in LMICs, Peru-specific normalization data are not currently available. We therefore used the Chilean EQ-5D value set to estimate QALYs, given cultural and economic similarities. However, it is unclear whether this value set adequately represents rural populations or the socioeconomic profile of our cohort. This limitation may partly explain the statistically significant difference in QALYs from the EQ-5D favoring the control group at 12 months. Additionally, the MID in EQ-5D-derived QALYs was derived from Uruguay, and its applicability to our study population remains uncertain. Third, LPG stoves and fuel were provided free of charge, and the effects on QOL and socioeconomic status may differ in real-world settings where households must purchase fuel. Finally, the absence of changes in reported monthly income may reflect local economic norms in which wealth is often accumulated through assets such as livestock or household infrastructure improvements rather than cash income, limiting the usefulness of income alone as an indicator of intervention impact [57].

## 5 | Conclusions

Although switching from biomass to LPG for cooking did not result in measurable improvements in QOL, it was associated with a perceived improvement in financial status. Future trials should evaluate these outcomes over longer follow-up periods, among populations with lower baseline QOL levels, using measurement instruments validated and normed to the local context.

## Figures and Tables

**FIGURE 1 |:**
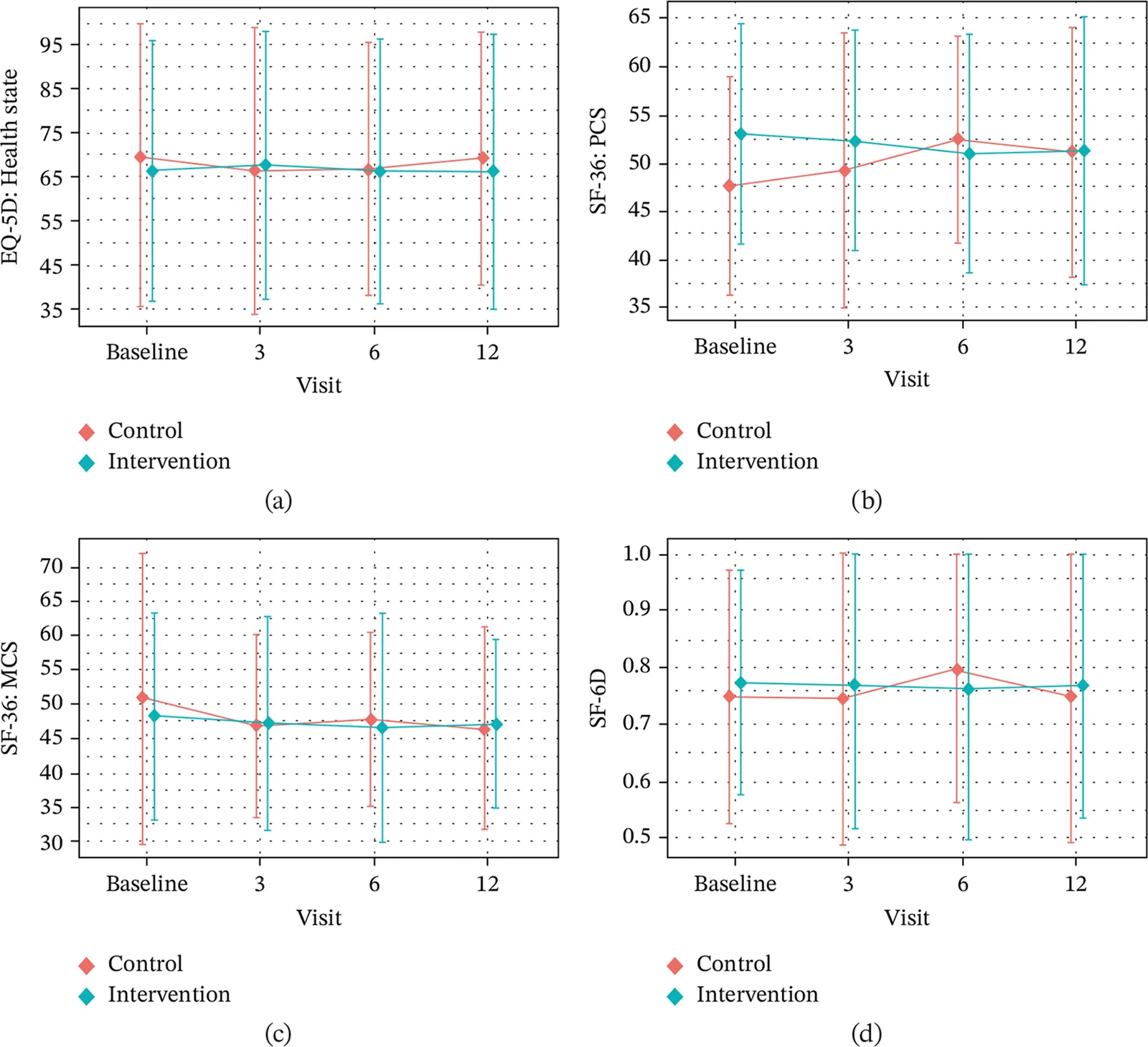
Mean scores for quality-of-life metrics by visit and trial assignment group in the Cardiopulmonary and Household Air Pollution (CHAP) trial. We plotted mean scores and 95% confidence intervals for quality-of-life metrics by visit and trial assignment group, with intervention participants shown in blue and controls in orange. At baseline, 3, 6, and 12 months, mean EQ-5D scores (a) for intervention vs. control participants were 66.6 vs. 69.8, 68.3 vs. 66.8, 66.5 vs. 67.0, and 67.0 vs. 71.7, respectively (*p* = 0.14); mean SF-36 physical component scores (b) were 53.1 vs. 47.6, 52.3 vs. 49.4, 51.0 vs. 52.5, and 51.3 vs. 51.2 (*p* = 0.52); mean SF-36 mental component scores (c) were 48.3 vs. 50.9, 47.3 vs. 46.9, 46.7 vs. 47.9, and 47.3 vs. 46.5 (*p* = 0.96); and mean SF-6D scores (d) were 0.77 vs. 0.75, 0.77 vs. 0.75, 0.76 vs. 0.80, and 0.77 vs. 0.75, respectively (*p* = 0.74).

**FIGURE 2 |:**
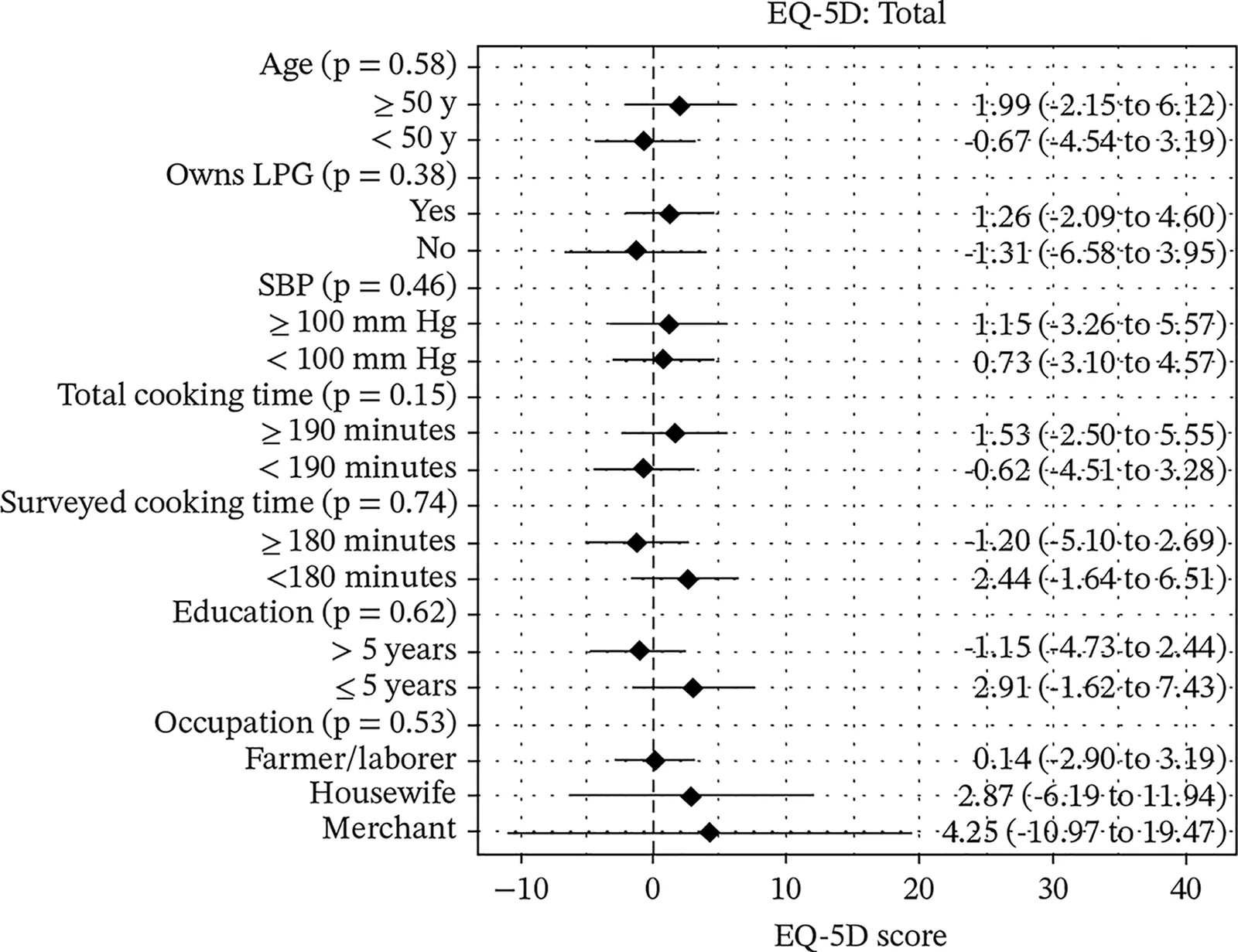
Subgroup analysis of between-arm differences in EQ-5D scores in the Cardiopulmonary and Household Air Pollution (CHAP) trial. We plotted the results of EQ-5D scores in subgroup analyses by age (≥ 50 vs. < 50 years), LPG ownership at baseline, systolic blood pressure (≥ 100 vs. < 100 mm Hg), total cooking time (≥ 190 vs. < 190 min), surveyed cooking time (≥ 180 vs. < 180 min), education (> 5 vs. ≤ 5 years), and occupation (farmer/laborer, housewife, and merchant). Estimated between-arm differences (intervention minus control) are represented by black diamonds, along with their corresponding 95% confidence intervals. The *p* value for each subgroup comparison is displayed next to the subgroup label on the right side of the figure.

**FIGURE 3 |:**
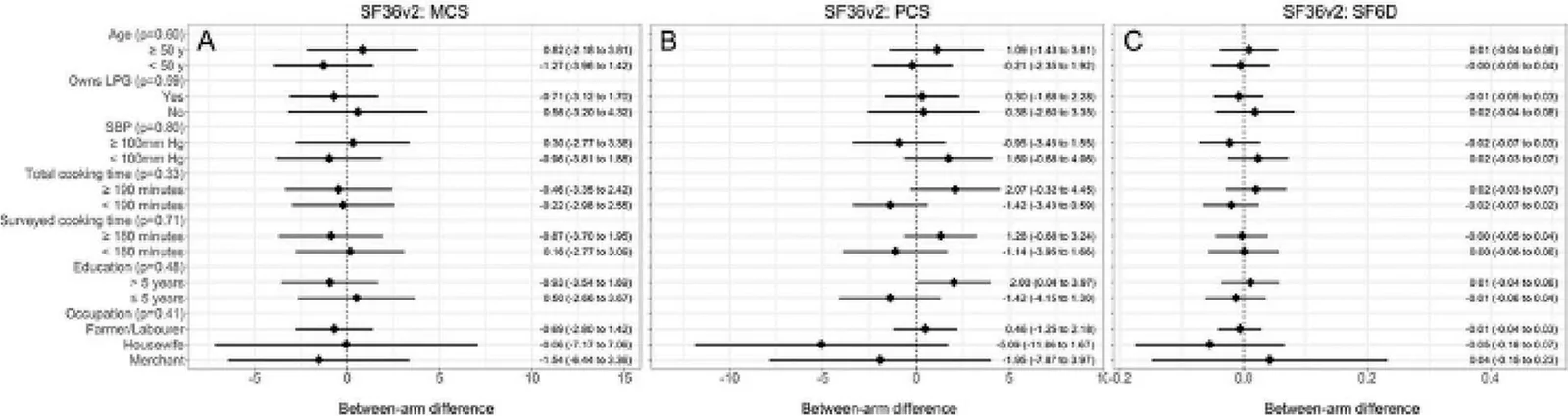
Subgroup analyses of between-arm differences in SF-36 Mental and Physical Component Scores and in the SF-6D score in the Cardiopulmonary and Household Air Pollution (CHAP) trial. We plotted subgroup analyses for SF-36 scores by age (≥ 50 vs. < 50 years), LPG ownership at baseline, systolic blood pressure (≥ 100 vs. < 100 mm Hg), total cooking time (≥ 190 vs. < 190 min), surveyed cooking time (≥ 180 vs. < 180 min), education (> 5 vs. ≤ 5 years), and occupation (farmer/laborer, housewife, and merchant). Estimated between-arm differences (intervention minus control) are represented by black diamonds, with corresponding 95% confidence intervals shown as lines. The *p* value for each subgroup comparison is provided next to the subgroup label. We plotted results for SF-36 Mental Component Score (A), results for the SF-36 Physical Component Score (B), and the SF-6D score (C).

**FIGURE 4 |:**
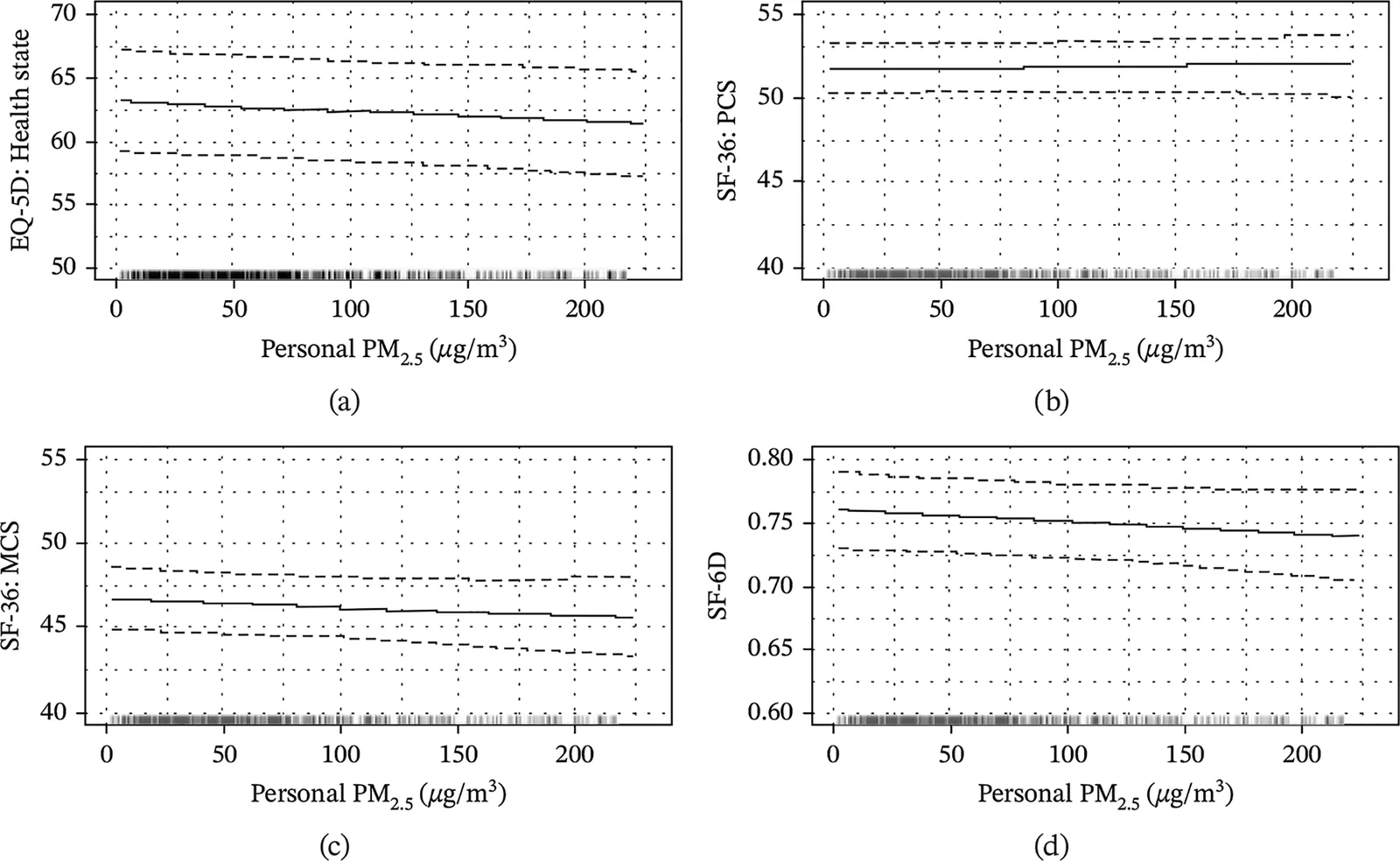
Adjusted associations between personal fine particulate matter (PM_2.5_) exposure and EQ-5D, SF-36, and SF-6D quality of life scores in the Cardiopulmonary and Household Air Pollution (CHAP) trial. Adjusted associations between fine particulate matter and quality of life measure (mean = solid line, 95% confidence interval = broken lines). Each plot is visually restricted to show up to the 95^th^ percentile of personal concentrations of PM_2.5_. We show a rug plot of the distributions of personal concentrations of PM_2.5_. Each panel corresponds to the association between PM_2.5_ and the EQ-5D (a), SF-36 Physical Component Score (b), SF-36 Mental Component Score (c), and SF-6D score (d).

**TABLE 1 |:** Baseline demographic, socioeconomic, anthropometric, and air pollution exposure characteristics of participants in the Cardiopulmonary and Household Air Pollution (CHAP) trial by assignment arm.

	Intervention (*n* = 90)	Control (*n* = 90)	*p* value*
Demographics			
Age (years), mean ± SD	48.6 ± 9.1	47.9 ± 11.1	0.59
Years of education, mean ± SD	6.1 ± 3.4	6.4 ± 3.3	0.61
Married or cohabitating, *n* (%)	78 (86.7%)	74 (82.2%)	0.54
Participant occupation: farmer, *n* (%)	78 (86.7%)	77 (85.6%)	0.68
Socioeconomics			
Wealth quintile, *n* (%)			
Poorest	51 (56.7%)	50 (55.6%)	0.37
Poor	32 (35.6%)	37 (41.1%)	
Middle	7 (7.8%)	3 (3.3%)	
Monthly income, *n* (%)			
0–99 soles (0–30 USD)	20 (22.2%)	25 (27.8%)	0.40
100–249 soles (30–75 USD)	33 (36.7%)	38 (42.2%)	
250–499 soles (76–151 USD)	34 (37.8%)	26 (28.9%)	
500 or more soles (152+ USD)	3 (3.3%)	1 (1.1%)	
Owned LPG at baseline, *n* (%)	64 (71.1%)	68 (75.6%)	0.61
Electricity in household, *n* (%)	85 (94.4%)	90 (100.0%)	0.07
Household owns a cell phone, *n* (%)	81 (90.0%)	81 (90.0%)	1.00
Owns 1 or more dogs, *n* (%)	66 (73.3%)	57 (63.3%)	0.20
Owns 1 or more pigs, *n* (%)	52 (57.8%)	54 (60.0%)	0.88
Minutes spent collecting fuel, mean ± SD	182.0 ± 157.1	151.0 ± 146.2	0.17
Minutes spent cooking assessed by survey, mean ± SD	198 ± 54	192 ± 54	0.51
Minutes spent cooking assessed by stove use monitor, mean ± SD	209 ± 62	214 ± 66	0.67
Anthropometry			
Height (cm), mean ± SD	150.8 ± 5.3	152.0 ± 4.7	0.11
Body mass index (kg/m^2^), mean ± SD	26.8 ± 4.2	26.9 ± 4.1	0.81
Personal exposures to household air pollution, mean ± SD			
Particulate matter ≤ 2.5 *μ*m (*μ*g/m^3^)	104.2 ± 99.7	126.4 ± 214.1	0.37
Black carbon (*μ*g/m^3^)	21.3 ± 22.3	19.1 ± 16.7	0.44
Carbon monoxide (ppm)	7.1 ± 8.4	6.6 ± 8.2	0.71
Nitrogen dioxide (ppb)	24.0 ± 18.4	22.0 ± 13.1	0.78

* We used *t*-tests to compare continuous variables and either chi-square tests or Fisher’s exact tests for categorical or dichotomous variables between groups.

**TABLE 2 |:** Baseline assessment of quality of life using the EQ-5D, SF-36, and SF-6D scores among participants in the Cardiopulmonary and Household Air Pollution (CHAP) trial by assignment arm.

	Intervention (*n* = 90)	Control (*n* = 90)	*p* value
EQ-5D-3L (*n* = 180)			
Total score, mean ± SD mean (SD)	66.5 (15.0)	69.8 (17.3)	0.18
Mobility, *n* (%)			
No problems	64 (71.1)	61 (67.8)	0.75
Some problems	26 (28.9)	29 (32.2)	
Extreme problems	0 (0)	0 (0)	
Self-care, *n* (%)			
No problems	87 (96.7)	85 (94.4)	0.72
Some problems	3 (3.3)	5 (5.6)	
Extreme problems	0 (0)	0 (0)	
Usual activities, *n* (%)			
No problems	79 (87.8)	77 (85.6)	0.83
Some problems	11 (12.2)	13 (14.4)	
Extreme problems	0 (0)	0 (0)	
Pain/discomfort, *n* (%)			
No problems	42 (46.7)	42 (46.7)	0.84
Some problems	47 (52.2)	46 (51.1)	
Extreme problems	1 (1.1)	2 (2.2)	
Anxiety/depression, *n* (%)			
No problems	63 (70.0)	56 (62.2)	0.35
Some problems	27 (30.0)	34 (37.8)	
Extreme problems	0 (0)	0 (0)	
Health state (quality-adjusted life years), mean ± SD	66.6 ± 15.0	69.8 ± 17.2	0.17
SF-36 Version 2 (*n = 16*)			
Physical health component, mean ± SD	53.1 ± 5.8	47.6 ± 5.7	0.08
Mental health component, mean ± SD	48.3 ± 7.7	50.9 ± 10.8	0.59
SF-6D (quality-adjusted life years), mean ± SD	0.8 ± 0.1	0.8 ± 0.1	0.66

**TABLE 3 |:** Self-reported economic situation, income changes, and monthly income at 12 months among participants in the Cardiopulmonary and Household Air Pollution (CHAP) trial by assignment arm.

	Control *(n* = 90)	Intervention (*n* = 89)	*p* value
Economic situation compared to before the intervention, *n* (%)			
A lot better	0 (0%)	1 (1%)	<0.01
A little better	12 (13%)	37 (42%)	
The same	75 (83%)	51 (57%)	
A little worse	3 (3%)	0 (%)	
Change in income compared to before the intervention, *n* (%)			
More	8 (9%)	29 (33%)	<0.01
Less	3 (3%)	0 (0%)	
The same	79 (88%)	60 (67%)	
Monthly income, *n* (%)			
0–99 soles (USD $0–33)	9 (10%)	4 (5%)	0.30
100–249 soles (USD $ 33–83)	35 (39%)	36 (40%)	
250–499 soles (USD $83–166)	39 (43%)	44 (49%)	
500 or more soles (USD $166+)	7 (8%)	5 (6%)	

## Data Availability

The data that support the findings of this study are available from the corresponding author upon reasonable request.
